# A somatization comorbidity phenotype impacts response to therapy in rheumatoid arthritis: post-hoc results from the certolizumab pegol phase 4 PREDICT trial

**DOI:** 10.1186/s13075-017-1412-z

**Published:** 2017-09-29

**Authors:** Jeffrey R. Curtis, Christopher Herrem, ’Matladi N. Ndlovu, Cathy O’Brien, Yusuf Yazici

**Affiliations:** 1 Division of Clinical Immunology and Rheumatology, University of Alabama at Birmingham, FOT 802, 510 20th Street South, Birmingham, AL 35294 USA; 2 Mylan, 1000 Mylan Blvd, Canonsburg, PA 15317 USA; 3 UCB Pharma, Allée de la Recherche 60, 1070 Brussels, Belgium; 4 NYU Hospital for Joint Diseases, New York University School of Medicine, 301 East 17th Street, New York, NY 10003 USA

**Keywords:** Rheumatoid arthritis, Disease activity, Patient-reported outcomes, Comorbidities, Depression, Anxiety, Fibromyalgia, RAPID3, CDAI

## Abstract

**Background:**

Comorbidities may contribute to disease activity and treatment response in rheumatoid arthritis (RA) patients. We defined a somatization comorbidity phenotype (SCP) and examined its influence on response to certolizumab pegol (CZP) using data from the PREDICT trial.

**Methods:**

Patients in PREDICT were randomized to the patient-reported Routine Assessment of Patient Index Data 3 (RAPID3) or physician-based Clinical Disease Activity Index (CDAI) for treatment response assessment. Post-hoc analyses identified patients with the SCP, which included diagnosis of depression, fibromyalgia/myalgias, and/or use of medications indicated for treatment of depression, anxiety, or neuropathic pain. The effect of the SCP on RAPID3 or CDAI response at week 12 and low disease activity (LDA; Disease Activity Score in 28 joints based on erythrocyte sedimentation rate ≤ 3.2) at week 52, in week-12 responders, was analyzed using non-parametric analysis of covariance (ANCOVA).

**Results:**

At baseline, 43% (313/733) of patients met the SCP classification. Patients with the SCP were 9% more likely to withdraw from the trial. American College of Rheumatology 20% (ACR20), ACR50, and ACR70 responses were 5–14% lower among those with the SCP, and 11% more patients reported adverse events (AEs). Patients without SCP in the CDAI arm were twice as likely to achieve LDA at week 52 compared with those with SCP (32% versus 16%). No differentiation by SCP was observed in the RAPID3 arm (pooled result 21.5%).

**Conclusions:**

We operationalized a potentially important somatization comorbidity phenotype in a trial setting that was associated with a substantially lower likelihood of treatment response and a higher frequency of AEs. Including large numbers of patients with this phenotype in RA trials may reduce the measured clinical effectiveness of a new molecule.

**Trial registration:**

ClinicalTrials.gov, NCT01255761. Registered on 6 December 2010.

**Electronic supplementary material:**

The online version of this article (doi:10.1186/s13075-017-1412-z) contains supplementary material, which is available to authorized users.

## Background

Comorbidities have been shown to substantially contribute to disease activity and treatment response in rheumatoid arthritis (RA) and should therefore be considered when devising treatment strategies for patients with RA [1–4]. Anxiety and depression are more common in people with RA than in the general population [5], and depression, fatigue, and fibromyalgia have been associated with increased disease activity, reduced odds for reaching clinical remission in RA, and reduced treatment effects and quality of life [4–8]. However, the impact of these comorbidities on the magnitude of response in a clinical trial setting has not been well characterized, and an efficient approach to identifying patients with these conditions within an RA trial program has not been previously well-described.

Clinical assessment tools, such as the Disease Activity Score in 28 joints (DAS28) and Clinical Disease Activity Index (CDAI), and patient-reported outcome-only tools, such as the Routine Assessment of Patient Index Data 3 (RAPID3), have proven effective for evaluating RA disease activity and predicting treatment response to guide patient management in RA [9–11]. The presence of comorbidities may also influence treatment outcomes indirectly due to the varying definitions of the state of remission or low disease activity (LDA) across different assessment tools [12]. There is currently no universal agreement on the best measurement tool for RA disease activity, given the strengths and limitations of each [9, 12–14].

The goal of these exploratory, post-hoc analyses was to define a “somatization comorbidity phenotype” (SCP) that could classify patients with RA participating in a clinical trial who had one or more conditions (e.g. fibromyalgia) that might have contributed to chronic pain independent of RA disease activity, or who had conditions (e.g. depression) other than RA that could have influenced pain and the patient’s self-management of their condition. The second aim was to evaluate whether this phenotype influenced treatment response as assessed by the CDAI and RAPID3 tools and more traditional measures (e.g. ACR response). The analysis was conducted in the Patient/Physician Reported Efficacy Determination in Clinical Practice (PREDICT) trial, among patients with moderate to severe RA initiating treatment with the anti-tumor necrosis factor (anti-TNF) agent, certolizumab pegol (CZP).

## Methods

### Study design

The PREDICT trial (NCT01255761) was a randomized, phase 4 trial that examined the predictability of CZP treatment success at week 52 based on treatment response at week 12, as assessed by the patient-reported RAPID3 or the mostly physician-based CDAI in patients with RA with moderate to severe, active disease. The primary hypotheses tested were whether the RAPID3 was comparable to the CDAI tool in assessing response to CZP therapy at week 12, and evaluating the positive predictive value of this classification against the RA disease state at week 52. Detailed methods and the primary results of the PREDICT study have been published previously [15].

In brief, patients aged ≥ 18 years, with a diagnosis of adult-onset RA for > 3 months’ duration at baseline were eligible for this 52-week study. RA was diagnosed according to the American College of Rheumatology (ACR) 1987 classification criteria [16]. Patients were also required to have an unsatisfactory response or intolerance to ≥ 1 disease-modifying antirheumatic drug (DMARD). Patients with fibromyalgia requiring treatment were excluded from the study as per protocol. The purpose of this exclusion was to remove patients who were hypothesized to have an attenuated treatment response as measured by the two tools (i.e. RAPID3 and CDAI) used in the trial, as both contain a patient measurement component. This exclusion was similar to a general exclusion in many RA trials where investigators are requested to exclude patients who have concomitant medical conditions that might interfere with the interpretation of the study results [17–19]. The exclusion was implemented by each local site investigator; no systematic identification or exclusion at the time of randomization was enforced.

Both RAPID3 and CDAI assessments were performed on all patients throughout the study at each visit. Patients were randomized 1:1 to protocolized management based upon either the RAPID3 or CDAI, and patients remained blinded throughout the study to their randomized group. Randomization was performed through an interactive voice/web-based response system (IXRS). All patients received a loading dose of CZP (400 mg at weeks 0, 2, and 4), followed by CZP 200 mg every second week (Q2W) until week 50.

At week 12, all patients were classified as responders or non-responders according to their randomized assessment group. RAPID3 response was defined as ≤ 6 (LDA) or ≥ 20% improvement from baseline, and CDAI response was defined as ≤ 10 (LDA) or ≥ 20% improvement from baseline. These cutoffs for CDAI and RAPID3 response were established in the PREDICT study protocol prior to published reports of a minimal clinically important difference (MCID) for CDAI [20] and RAPID3 [21]. All patients who were not week-12 RAPID3 or CDAI responders, or were withdrawn prior to week 12, were classified as non-responders. The non-responders included a subset of patients who had no improvement by week 12 (“week-12 failures”; those with no RAPID3 improvement or < 1 point CDAI improvement, based on the assessment group to which they were randomized), and were withdrawn from the study and CZP treatment. All other non-responders who were not week-12 failures continued treatment with CZP 200 mg Q2W, unless they reached a high disease activity state (CDAI > 22 or RAPID3 > 12) [22] at two consecutive visits, at which point they were withdrawn from the study.

### Somatization comorbidity phenotype

The somatization comorbidity phenotype (SCP) was defined post hoc to the trial, but a priori to conducting these analyses. Based on clinical interest and suggestions from observational data that fibromyalgia, depression, anxiety, psychological distress and similar conditions might influence RA assessment [17, 23–26], we hypothesized that patients with this phenotype might have a suboptimal treatment response in an RA clinical trial. The phenotype was operationally defined by use of concomitant medications indicated for the treatment of depression, anxiety, or neuropathic pain (i.e. selective serotonin reuptake inhibitors (SSRIs), duloxetine, venlafaxine, milnacipran, pregabalin, gabapentin, amitriptyline, tizanidine, cyclobenzaprine, methocarbamol, or metaxalone), or ongoing baseline medical diagnosis of depression, chronic pain, fibromyalgia, or myalgias (classified using the *Medical Dictionary for Regulatory Activities* (MedDRA) version 15.1 with the terms: cervical tension myalgia, generalized muscle pain; muscle pain; muscle pain hip area; myalgia; and myalgias). Information classifying patients by SCP status was taken from the medical history and concomitant medication data taken at the beginning of the study. Data on the duration of medical diagnoses were not collected, therefore there was no requirement for the use of these concomitant medications or diagnoses to be of a chronic nature. A diagnosis of insomnia and use of narcotics was not included in the SCP definition, given that RA-related symptoms might commonly affect sleep, and RA-specific pain may be treated with narcotics.

### Statistical analysis

All data were analyzed post hoc and the full analysis set was used, which included all patients who had a valid baseline efficacy measurement and at least one valid post-baseline efficacy measurement. The RAPID3 and CDAI arms were stratified by SCP status. RAPID3/CDAI response at week 12 and the DAS28 (based on erythrocyte sedimentation rate (ESR)) LDA (defined as ≤ 3.2) at week 52, including 95% confidence intervals (CIs) of the difference in response rates between groups, were analyzed using non-parametric analysis of covariance (ANCOVA) [27–29], with assessment tool (RAPID3 or CDAI) or SCP status (plus SCP or minus SCP) as a factor and baseline DAS28(ESR) score, gender, age, prior anti-TNF use, and duration of RA (<2 or ≥ 2 years) as covariates. Imputation for missing data was based on non-responder imputation for dichotomous variables, and last observation carried forward (LOCF) for continuous variables.

The safety set consisted of all enrolled patients who received at least one dose of study medication, with treatment-emergent adverse events (AEs) defined as taking place at any time between the first dose and 70 days after the last dose of study drug. All AEs were classified by primary system organ class (SOC), using MedDRA version 15.1. Incidence rates (IRs) were calculated per 100 patient-years (PY), with 95% CIs. Time at risk was measured from initiation of CZP up to the occurrence of the first serious infectious event (SIE), or the total time at risk for patients without SIEs (up to 70 days after the last study dose or patient withdrawal). All statistical analyses were performed in SAS® software (SAS Institute, Cary, NC, USA), version 9.1.3 or later.

## Results

### Patient characteristics

**Fig. 1:**
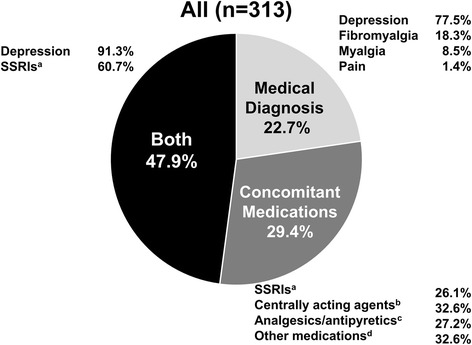
Proportions of patients meeting criteria for the somatization comorbidity phenotype classification. Full analysis set. The denominator used to calculate the percentage for each category was based upon the total number of patients within the comorbidity/treatment assignment category; the sum of percentages was over 100% due to some patients taking more than one concomitant medication and/or possessing more than one medical diagnosis. ^a^Defined as Anatomical Therapeutic Chemical Classification System (ATC) code N06AB. ^b^Defined as ATC code M03BX. ^c^Defined as ATC code N02BG. ^d^Included medications in ATC codes N06AA (non-selective monoamine reuptake inhibitors), N06AX (other antidepressants), M03BA (carbamic acid esters), and M03BB (oxazol, thiazine, and triazine derivatives). SSRI selective serotonin reuptake inhibitors

The full analysis set included 733 patients, with 368 patients randomized to RAPID3 and 365 patients randomized to the CDAI arm of the study. A total of 313 patients (43% overall; RAPID3, n = 151; CDAI, n = 162) met the SCP classification criteria at study baseline. Of these, 92 patients (29.4%; RAPID3, n = 47; CDAI, n = 45) met the SCP classification due to concomitant medications only (predominantly use of central acting agents (i.e. baclofen, tizanidine, cyclobenzaprine) or SSRIs); 71 patients (22.7%; RAPID3, n = 33; CDAI, n = 38) met the phenotype due to medical diagnoses only (predominantly depression). The remaining 150 patients (47.9%; RAPID3, n = 71; CDAI, n = 79) had both concomitant medications and a medical diagnosis (e.g. SSRIs, plus a diagnosis of depression) (Fig. 1, Additional file 1: Table S1).

Baseline characteristics including CDAI, RAPID3, Patient’s Global Assessment of Disease Activity (PtGADA), Multi-Dimensional Health Assessment Questionnaire (MDHAQ) Pain, and physical function scores were relatively comparable between patients with and without the phenotype (+SCP and −SCP) (Table 1). A similar proportion of +SCP patients (7.9%, 12/151 RAPID3 and 7.4%, 12/162 CDAI assessed) and −SCP patients (10.6%, 23/217 RAPID3 and 7.4%, 15/203 CDAI assessed) were withdrawn from the study by week 12 due to lack of efficacy. However, a larger proportion of +SCP patients (16.5%, 34/206) than −SCP patients (7.6%, 22/290) were withdrawn after week 12 due to lack of efficacy, a 9% difference between groups (Additional file 1: Table S2).

**Table 1:** Patient baseline characteristics by SCP status^a^

	+SCP (n = 313)	−SCP (n = 420)
Mean (SD), *n* unless otherwise stated		
Randomized to assessment arm		
RAPID3, *n* (% overall)	151 (48.2)	217 (51.7)
CDAI, *n* (% overall)	162 (51.8)	203 (48.3)
DAS28(ESR)	6.34 (1.07), 306	6.27 (1.07), 411
CDAI	41.41 (13.25), 307	39.30 (13.01), 416
RAPID3	16.76 (5.31), 308	15.56 (5.73), 415
TJC	16.82 (6.81), 308	15.08 (6.74), 416
SJC	12.12 (5.65), 308	12.26 (5.66), 416
PhGADA (VAS)^b^	6.19 (1.83), 308	6.21 (1.78), 416
PtGADA (VAS)^b^	6.21 (2.23), 308	5.74 (2.35), 416
Pain MDHAQ-PN (0–10 scale)	6.83 (2.07), 308	6.33 (2.16), 415
Physical function MDHAQ-FN (converted to 0–10 scale for RAPID3)	3.73 (1.82), 308	3.49 (1.92), 416

Full analysis set of the PREDICT trial. For these baseline values, some patients (<3% overall) were excluded due to prohibited concomitant medications affecting their assessments. Those with valid assessments at subsequent study visits were not excluded from the overall study

*Abbreviations*: *SCP* somatization comorbidity phenotype, *ESR* erythrocyte sedimentation rate, *CDAI* Clinical Disease Activity Index, *DAS28* Disease Activity Score in 28 joints, *MDHAQ* Multidimensional Health Assessment Questionnaire, *PhGADA* Physician’s Global Assessment of Disease Activity, *PtGADA* Patient’s Global Assessment of Disease Activity, *RAPID3* Routine Assessment of Patient Index Data 3, *SJC* swollen joint count, *TJC* tender joint count

^a^Combining the RAPID3 and CDAI arms

^b^Visual analog scale (0–100 mm converted to cm)

### Disease activity control and response at week 12

**Fig. 2:**
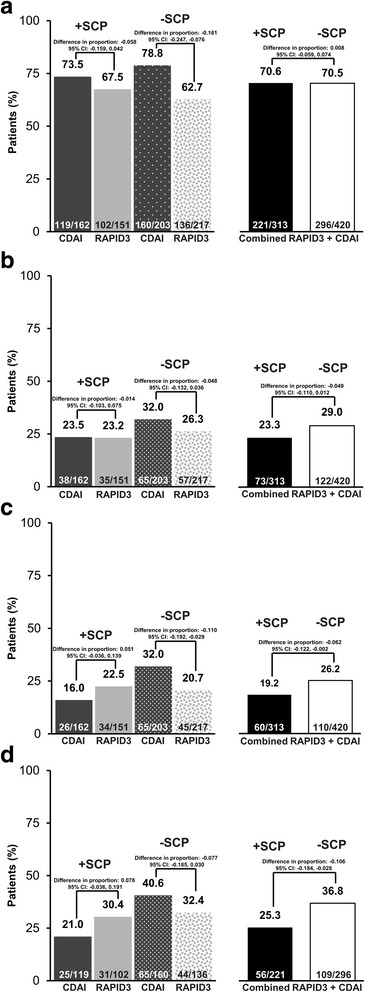
Clinical response by assessment tool and presence of somatization comorbidity phenotype (SCP). **a** Proportion of Routine Assessment of Patient Index Data 3 (RAPID3) and Clinical Disease Activity Index (CDAI) responders^a^ at week 12. **b** Proportion of patients assigned to RAPID3 and CDAI with Disease Activity Score in 28 joints based on erythrocyte sedimentation rate (DAS28(ESR)) low disease activity (LDA) at week 12. **c** Proportion of patients with DAS28(ESR) LDA at week 52. **d** Proportion of RAPID3 and CDAI week 12 responders^a^ with DAS28(ESR) LDA at week 52. Full analysis set. LDA was defined as DAS28(ESR) ≤ 3.2. ^a^RAPID3 response was defined as ≤ 6 or 20% improvement from baseline, and CDAI response was defined by ≤ 10 or 20% improvement from baseline. Missing data were handled by non-responder imputation

The proportion of patients classified as responders at week 12 according to RAPID3 or CDAI definitions was similar, irrespective of SCP status (+SCP 70.6%, 221/313; −SCP 70.5%, 296/420; difference in proportion 0.8% (95% CI − 5.9%, 7.4%)) (Fig. 2a). However, in the CDAI group, the likelihood of being classified as a week-12 responder was incrementally smaller for +SCP patients (73.5%, 119/162) versus −SCP patients (78.8%, 160/203) (Fig. 2a). Combining the RAPID3 and CDAI arms, fewer +SCP patients (23.3%, 73/313) than −SCP patients achieved DAS28(ESR) LDA at week 12 (29.0%, 122/420; difference in proportion − 4.9% (95% CI − 11.0%, 1.2%)) (Fig. 2b).

### Disease activity control and response at week 52

At week 52, only half as many +SCP patients (16.0%, 26/162) in the CDAI assessment group achieved LDA, in comparison with the −SCP patients (32.0%, 65/203) (Fig. 2c). Similarly, only about half of the +SCP patients (21.0%, 25/119) in the CDAI assessment group who were week-12 responders achieved LDA at week 52 in comparison to the −SCP patients (40.6%, 65/160) (Fig. 2d). In contrast, this phenotype was less differentiating among patients randomized to the RAPID3 assessment arm (Fig. 2c, d). Overall, a smaller proportion of +SCP patients (19.2%, 60/313) achieved LDA at week 52 than −SCP patients (26.2%, 110/420; difference in proportion − 6.2% (95% CI − 12.2%, − 0.2%)) (Fig. 2c). Amongst the week 12 responders, fewer +SCP patients (25.3%, 56/221) achieved LDA at week 52 in comparison with the −SCP patients (36.8%, 109/296; difference in proportion − 10.6% (95% CI − 18.4%, − 2.8%–)) (Fig. 2d).

The improvement in mean DAS28(ESR) scores was consistently better in −SCP patients throughout the duration of the study. The difference according to SCP status was most apparent for patients randomized to the CDAI assessment group (Additional file 1: Figure S1). At week 52, the median DAS28(ESR) score for week-12 responders was 3.97 for the 221 +SCP patients, versus 3.37 for the 296 −SCP patients, equivalent to a 0.6-unit difference between presence and absence of the SCP. Similar trends were observed for RAPID3 and CDAI, where +SCP patients reported higher DAS28(ESR) scores (RAPID3 9.06 ± 6.19, CDAI 15.34 ± 13.07 (mean ± SD)) than −SCP patients (RAPID3 6.99 ± 6.10, CDAI 11.17 ± 11.41).

### Clinical response by achievement of ACR20/50/70

**Fig. 3:**
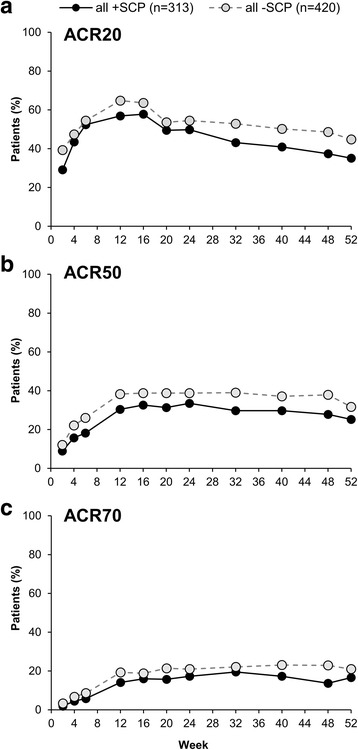
American College of Rheumatology (ACR) response rates by somatization comorbidity phenotype (SCP) status. **a** ACR20. **b** ACR50. **c** ACR70. Full analysis set. Missing data were handled by non-responder imputation

Overall, approximately 5–10% fewer +SCP patients achieved ACR 20% response (ACR20), ACR50, and ACR70 throughout the study. At week 52, 44.8% (188/420), 31.7% (133/420), and 21.0% (88/420) of −SCP patients achieved ACR20, ACR50, and ACR70, respectively, versus 35.1% (110/313), 25.2% (79/313), and 16.6% (52/313) of +SCP patients (Fig. 3a–c).

**Fig. 4:**
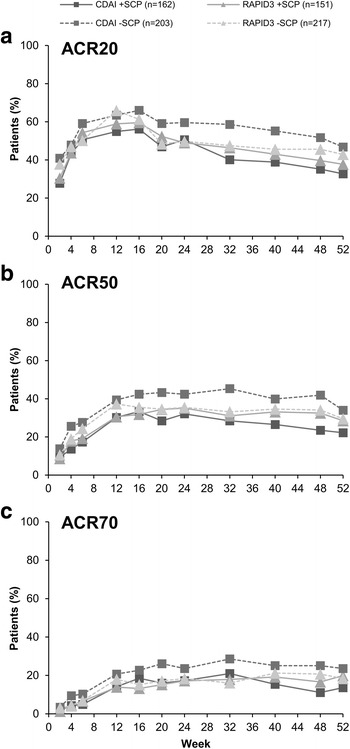
American College of Rheumatology (ACR) response rates by assessment group and somatization comorbidity phenotype (SCP) status. **a** ACR20. **b** ACR50. **c** ACR70. Full analysis set. Missing data were handled by non-responder imputation

This trend was particularly noticeable in the group assessed by CDAI, with 46.8% (95/203), 34.0% (69/203), and 23.6% (48/203) of −SCP patients versus 32.7% (53/162), 22.2% (36/162), and 13.6% (22/162) of +SCP patients achieving ACR20, ACR50, and ACR70, respectively, at week 52. These results indicate an approximate 10–14% lower ACR clinical response in +SCP patients (Fig. 4a–c). Smaller differences in ACR20, ACR50, and ACR70 response rates were observed for those patients randomized to RAPID3 (Fig. 4a–c).

### Safety results

The safety set comprised a total of 736 patients. Overall, the cumulative one-year incidence of treatment-emergent adverse events (TEAEs) was higher in +SCP patients (82.5%, 259/314) versus −SCP patients (71.1%, 300/422), with an incidence rate (IR) of 351.3/100 PY for +SCP patients versus 201.9/100 PY for −SCP patients. In particular, the incidence of infections and infestations was higher in +SCP patients compared to −SCP patients (108.6/100 PY versus 56.0/100 PY) (Table 2). Similarly, the IR of serious infectious events (SIEs) was also higher in +SCP patients compared with −SCP patients (5.0/100 PY versus 1.7/100 PY, a difference of 3.3/100 PY). The number of study discontinuations due to AEs was similar among +SCP and −SCP patients.

**Table 2:** Summary of adverse events by SCP status^a^

Number (percentage), incidence ratio (95% CI)^b^, unless otherwise indicated	+SCP (n = 314)	−SCP (n = 422)	All patients (n = 736)
Total time at risk (100 PY)	2.44	3.49	5.92
Any TEAEs	259 (82.5), 351.3 (309.8, 396.8)	300 (71.1), 201.9 (179.7, 226.1)	559 (76.0), 251.4 (231.0, 273.2)
TEAEs (≥5% in any SOC^c^)			
Gastrointestinal disorders	65 (20.7), 31.3 (24.2, 39.9)	73 (17.3), 24.0 (18.8, 30.2)	138 (18.8), 26.9 (22.6, 31.8)
General disorders and administration site conditions	48 (15.3), 22.5 (16.6, 29.8)	53 (12.6), 17.1 (12.8, 22.3)	101 (13.7), 19.3 (15.7, 23.4)
Infections and infestations	166 (52.9), 108.6 (92.7, 126.4)	144 (34.1), 56.0 (47.2, 65.9)	310 (42.1), 75.6 (67.4, 84.5)
Injury, poisoning, and procedural complications	41 (13.1), 18.6 (13.4, 25.3)	34 (8.1), 10.2 (7.1, 14.3)	75 (10.2),13.6 (10.7, 17.0)
Investigations	39 (12.4), 17.6 (12.5, 24.1)	50 (11.8), 15.8 (11.7, 20.8)	89 (12.1), 16.5 (13.3, 20.4)
Metabolism and nutrition disorders	21 (6.7), 9.1 (5.6, 13.9)	22 (5.2), 6.6 (4.1, 9.9)	43 (5.8), 7.6 (5.5, 10.2)
Musculoskeletal and connective tissue disorders	100 (31.8), 52.5 (42.7, 63.9)	80 (19.0), 26.0 (20.6, 32.4)	180 (24.5), 36.2 (31.1, 41.8)
Nervous system disorders	59 (18.8), 28.5 (21.7, 36.8)	34 (8.1), 10.3 (7.1, 14.3)	93 (12.6), 17.3 (14.0, 21.2)
Psychiatric disorders	29 (9.2), 12.8 (8.5, 18.3)	10 (2.4), 2.9 (1.4, 5.4)	39 (5.3), 6.9 (4.9, 9.4)
Respiratory, thoracic, and mediastinal disorders	48 (15.3), 22.4 (16.5, 29.7)	48 (11.4), 14.9 (11.0, 19.8)	96 (13.0), 17.9 (14.5, 21.9)
Skin and subcutaneous tissue disorders	44 (14.0), 20.1 (14.6, 27.0)	50 (11.8), 15.5 (11.5, 20.5)	94 (12.8), 17.4 (14.0, 21.3)
Vascular disorders	26 (8.3), 11.4 (7.5, 16.8)	17 (4.0), 5.0 (2.9, 8.0)	43 (5.8), 7.6 (5.5, 10.2)
Serious TEAEs	34 (10.8), 14.8 (10.3, 20.7)	37 (8.8), 10.9 (7.7, 15.0)	71 (9.6), 12.5 (9.8, 15.8)
Serious infections and infestations	12 (3.8), 5.0 (2.6, 8.7)	6 (1.4), 1.7 (0.6, 3.8)	18 (2.4), 3.1 (1.8, 4.8)
Discontinuation due to TEAEs^d^	34 (10.8), NA	44 (10.4), NA	78 (10.6), NA
Drug-related TEAEs	80 (25.5), NA	93 (22.0), NA	173 (23.5), NA
Severe TEAEs	38 (12.1), NA	38 (9.0), NA	76 (10.3), NA
Deaths (TEAEs leading to death)	1 (0.3), NA	1 (0.2), NA	2 (0.3), NA

Safety set. *Medical Dictionary for Regulatory Activities* (MedDRA) v15.1

*SCP* somatization comorbidity phenotype, *TEAEs* treatment-emergent adverse events, *PY* patient-years, *NA* data not available

^a^Combining the RAPID3 and CDAI arms.

^b^n: number of subjects reporting at least one TEAE in that category, incidence rates reported per 100 patient-years

^c^System organ class

^d^There was one patient in this group reporting a TEAE of pneumonia, who did not discontinue the study drug permanently

## Discussion

In this large RA population with moderate to severe, active RA, we have described a method to efficiently identify a potentially important patient phenotype in a clinical trial setting that includes depression, anxiety, and chronic pain syndromes such as fibromyalgia and neuropathic pain. Patients enrolled in the PREDICT study were assigned to this phenotype based on concomitant medications and/or medical diagnosis at baseline. In total, 43% of patients met the SCP classification at study baseline, and patients with this phenotype demonstrated a substantially lower likelihood of treatment response.

In particular, the SCP that we have defined appears to be important with respect to both predicting treatment response at week 12 (23.3% of +SCP patients achieved DAS28(ESR) LDA versus 29.0% of −SCP patients) and the likelihood of achieving an LDA state after one year of treatment with CZP (25.3% of +SCP patients versus 36.8% of −SCP patients). Furthermore, +SCP patients were 5–14% less likely to achieve ACR20, ACR50, and ACR70 throughout the duration of the 52-week study. Those with the SCP were also 9% more likely to withdraw over the course of this one-year trial. Although speculative, this higher rate of withdrawal for patients with the SCP could be due to perceived lack of benefit of CZP, lower adherence, a higher rate of some AEs, or other factors.

Overall, when using CDAI as the assessment tool, patients with the SCP appeared less likely to be classified as treatment responders by week 12. Response rates appeared to be more comparable regardless of SCP status when the RAPID3 tool was used. This may reflect the fact that CDAI and the DAS28(ESR) – the primary outcome used in the PREDICT trial – are more highly correlated with each other than the RAPID3 and the DAS28(ESR) [30–32].

Our results may have implications for routine clinical care in deciding which tool to use for RA assessment, given that the RAPID3 reflects a more patient-centric and arguably more holistic assessment than the CDAI, and it is also likely to be easier to implement in busy practices [33]. Irrespective of the SCP phenotype, the primary results of the PREDICT trial showed some differences between the CDAI versus the RAPID3 in the proportion of people classified as responders at 12 weeks [15]. Ultimately, the choice of the preferred tool to assess RA disease activity and treatment response for patients both with and without the SCP phenotype must balance multiple considerations related to performance, feasibility, and the relative importance of physician versus patient data.

The results of our study align with data from previous reports, which demonstrate that depression, anxiety, fibromyalgia, and neuropathic pain are prevalent comorbidities in patients with RA and are associated with increased disease activity, poor treatment adherence, and a reduced likelihood of achieving LDA or remission [2, 6, 7, 24, 34, 35]. Ours is among the first to quantify the impact on a large RA clinical trial patient population with active disease starting treatment with a therapeutic agent known to be effective based on the results of previous phase 3 studies. These differences extended not only to the clinical efficacy outcomes, but also to safety endpoints. The incident rate of TEAEs was higher in +SCP patients than in −SCP patients, with a notably higher rate of infectious events in the +SCP group, including SIEs. The magnitude of the difference in the rates of SIEs between +SCP and −SCP patients (3.3/100 PY) in this analysis, all of whom were receiving CZP therapy, was comparable to the difference between CZP and placebo treatment in other RA studies [36]. Depression and other patient factors have been associated with adverse events such as infections [37], cardiovascular disease [38], myocardial infarction [39], and an increased risk of mortality [40], according to past studies. This may reflect a true biologic association, as inflammatory markers such as C-reactive protein have been shown to be associated with depression [41], although these conditions may be a proxy for related factors (e.g. health-seeking behavior, perceived illness severity) that could impact the reporting of more subjective or less severe adverse events. Collectively, these findings suggest that the SCP phenotype may be a useful and easily ascertained proxy in an RA clinical trial population for factors making patients less likely to achieve a good clinical response and less likely to tolerate some RA treatments.

The limitations of these analyses included the fact that the PREDICT study only enrolled patients in the USA, and the patients with fibromyalgia symptomatic enough to be treated were excluded by protocol at the discretion of the local investigator, though no systematic identification or exclusion at the time of randomization was enforced. In addition, it is possible that patients with the SCP but who did not have a formal diagnosis and who were not taking any indicated medications, were misclassified. Assuming that the patients with the most severe manifestations of this condition were excluded from the study, and that some patients had not been diagnosed nor were using medication to manage their SCP, our results likely represent a conservative estimate of the effect that the SCP phenotype had on treatment response as measured by standard tools such as the achievement of DAS28(ESR) LDA and ACR response, suggesting that the impact of this phenotype in real-world settings may be even more profound. This exclusion might raise concerns about the generalizability of the study’s results to the typical RA patient population seen in the USA. However, even with the exclusion in place, we note that a high proportion of patients using medications indicated for depression, anxiety, neuropathic pain, and fibromyalgia were in fact enrolled in the study and had the SCP (43% of the PREDICT trial population). Moreover, in many other RA trials, study criteria commonly exclude patients with conditions deemed by the site investigator “to be symptomatic enough to interfere with the measurement of treatment response” [19, 42]. However, this exclusion criterion is highly subjective and is rarely enforced in a reproducible fashion. For this reason, RA trials can be expected to include a mix of these patients, but previously these individuals have not been easily identified without the added burden of incorporating additional, specific screening tools to classify patients as having fibromyalgia [43] or depression [44]. While these types of screening instruments could be systematically applied to an RA cohort or trial [45], the phenotype we defined has the advantage that it can be applied to data routinely captured at baseline in RA clinical trials, requiring only concomitant medications and/or diagnoses instead of a specific screening instrument, thereby minimizing participant burden for additional data collection.

These analyses have highlighted that the inclusion of large numbers of patients with the phenotype in an RA trial may appreciably affect the proportion of patients who are able to achieve an absolute disease state treatment target, such as LDA or remission. On the other hand, if new trials excluded individuals with the somatization comorbidity phenotype from a trial, this could somewhat limit the generalizability of the results and hinder comparability between trials, as the proportion of such patients in RA trials across the globe is likely to vary widely. In addition, allowing patients with the somatization comorbidity phenotype in RA studies may contribute to a ceiling effect, as patients may not demonstrate as much improvement with effective RA therapies in comparison to patients without this phenotype.

## Conclusions

In the PREDICT clinical trial, 43% of patients met the “somatization comorbidity phenotype” classification criteria for diagnosis and/or use of medications indicated for depression, fibromyalgia, anxiety, or neuropathic pain. These patients were significantly more likely to withdraw from the trial and/or experience adverse events, and approximately 10–14% less likely to achieve a clinical response. These results suggest that depending on the outcome measure used (e.g. DAS28), enrolling large numbers of patients with this phenotype may affect the proportion able to achieve LDA or remission, making it advisable to consider whether to include, identify, or stratify these patients during the study design or study screening processes in future RA clinical trials.

## Additional file

Additional file 1:Table S1. SCP categories and medical history/diagnoses at baseline, with missing data handled by non-responder imputation (NRI). **Table S2.** Patients withdrawn from the study at/before and after week 12 due to lack of efficacy. **Figure S1.** Mean DAS28(ESR) score by SCP status, with missing data handled by last observation carried forward (LOCF). Full analysis set. (DOCX 1302 kb)

## Abbreviations

ACR: American College of Rheumatology
AE: Adverse event
ANCOVA: Analysis of covariance
CDAI: Clinical Disease Activity Index
CI: Confidence interval
CZP: Certolizumab pegol
DAS: Disease activity score
DMARD: Disease-modifying anti-rheumatic drug
ESR: Erythrocyte sedimentation rate
IR: Incidence rate
IXRS: Interactive voice/web-based response system
LDA: Low disease activity
LOCF: Last observation carried forward
MCID: Minimal clinically important difference
MDHAQ: Multi-Dimensional Health Assessment Questionnaire
MedDRA: Medical Dictionary for Regulatory Activities
NMSC: Non-melanoma skin cancer
PhGADA: Physician's Global Assessment of Disease Activity
PtGADA: Patient's Global Assessment of Disease Activity
PY: Patient years
Q2W: Every 2 weeks
RA: Rheumatoid arthritis
RAPID3: Routine Assessment of Patient Index Data 3
SCP: Somatization comorbidity phenotype
SD: Standard deviation
SIE: Serious infectious event
SJC: Swollen joint count
SOC: System organ class
SSRIs: Selective serotonin reuptake inhibitors
TEAE: Treatment-emergent adverse event
TNF: Tumor necrosis factor
