# Supplementary material for: A somatization comorbidity phenotype impacts response to therapy in rheumatoid arthritis: post-hoc results from the certolizumab pegol phase 4 PREDICT trial
Source: Arthritis Res Ther. 2017 Sep 29;19:215. doi: 10.1186/s13075-017-1412-z (PMC5622491; doi:10.1186/s13075-017-1412-z)
Supplement: Additional file 1:Table S1. — SCP categories and medical history/diagnoses at baseline, with missing data handled by non-responder imputation (NRI). Table S2. Patients withdrawn from the study at/before and after week 12 due to lack of efficacy. Figure S1. Mean DAS28(ESR) score by SCP status, with missing data handled by last observation carried forward (LOCF). Full analysis set. (DOCX 1302 kb) [file 13075_2017_1412_MOESM1_ESM.docx]

Additional file 1

Table S1. SCP categories and medical history/diagnoses at Baseline (NRI)

| Comorbidity category  Medical history/diagnoses, n (%)^a^ | RAPID3 (n=151) | CDAI  (n=162) | All patients with the SCP  (N=313) |
| --- | --- | --- | --- |
| Concomitant medications  SSRIs^b^  Centrally acting agents^c^  Analgesics/antipyretics^d^  Other medications^e^ | **47 (31.1)**  18 (38.3)  16 (34.0)  11 (23.4)  15 (31.9) | **45 (27.8)**  6 (13.3)  14 (31.1)  14 (31.1)  15 (33.4) | **92 (29.4)**  24 (26.1)  30 (32.6)  25 (27.2)  30 (32.6) |
| Medical diagnosis  Depression  Fibromyalgia  Myalgia  Pain | **33 (21.9)**  26 (78.8)  7 (21.2)  3 (9.1)  0 | **38 (23.5)**  29 (76.3)  6 (15.8)  3 (7.9)  1 (2.6) | **71 (22.7)**  55 (77.5)  13 (18.3)  6 (8.5)  1 (1.4) |
| Both  SSRIs^b^  Centrally acting agents^c^  Analgesics/antipyretics^d^  Depression  Fibromyalgia  Myalgia  Other medications^e^ | **71 (47.0)**  53 (74.6)  12 (16.9)  9 (12.7)  66 (93.0)  12 (16.9)  1 (1.4)  22 (31.0) | **79 (48.8)**  38 (48.1)  17 (21.5)  13 (16.5)  71 (89.9)  13 (16.5)  4 (5.1)  41 (52.0) | **150 (47.9)**  91 (60.7)  29 (19.3)  22 (14.7)  137 (91.3)  25 (16.7)  5 (3.3)  63 (42.0) |

Full analysis set. ^a^The denominator used to calculate the percentage for each category was based upon the total number of patients within the comorbidity/treatment assignment category; the sum of percentages was over 100% due to some patients taking more than one concomitant medication and/or possessing more than one medical diagnosis; ^b^Defined as Anatomical Therapeutic Chemical Classification System (ATC) code N06AB; ^c^Defined as ATC code M03BX; ^d^Defined as ATC code N02BG; ^e^Included medications in ATC codes N06AA (non-selective monoamine reuptake inhibitors), N06AX (other antidepressants), M03BA (carbamic acid esters), and M03BB (oxazol, thiazine, and triazine derivatives).

**Table S2. Patients withdrawn from the study at/before and after Week 12 due to lack of efficacy**

| n/N (%) | RAPID3 (n=368) | | CDAI (n=365) | | Overall (n=733) | |
| --- | --- | --- | --- | --- | --- | --- |
|  | **+SCP** | **-SCP** | **+SCP** | **-SCP** | **+SCP** | **-SCP** |
| At/before Week 12^a^ | 35/368 (9.5) | | 27/365 (7.4) | | 62/733 (8.5) | |
|  | 12/151 (7.9) | 23/217 (10.6) | 12/162 (7.4) | 15/203 (7.4) | 24/313 (7.7) | 38/420 (9.0) |
| Any through Week 52^b^ | 34/237 (14.3) | | 22/259 (8.5) | | 56/496 (11.3) | |
|  | 20/101 (19.8) | 14/136 (10.3) | 14/105 (13.3) | 8/154 (5.2) | 34/206 (16.5) | 22/290  (7.6) |
|  |  |  |  |  | Difference in proportion: 0.080 [95% CI: 0.022, 0.139] | |

^a^IXRS withdrawals, non-responders, or otherwise did not qualify to continue; ^b^Post-week 12 withdrawal criteria based on randomized tool, High Disease Activity (RAPID3 >12 in the RAPID3 arm or CDAI >22, in the CDAI arm) at two consecutive visits after Week 12 (i.e., Weeks 16, 20, 24, 32, 40, 48, 52).

**
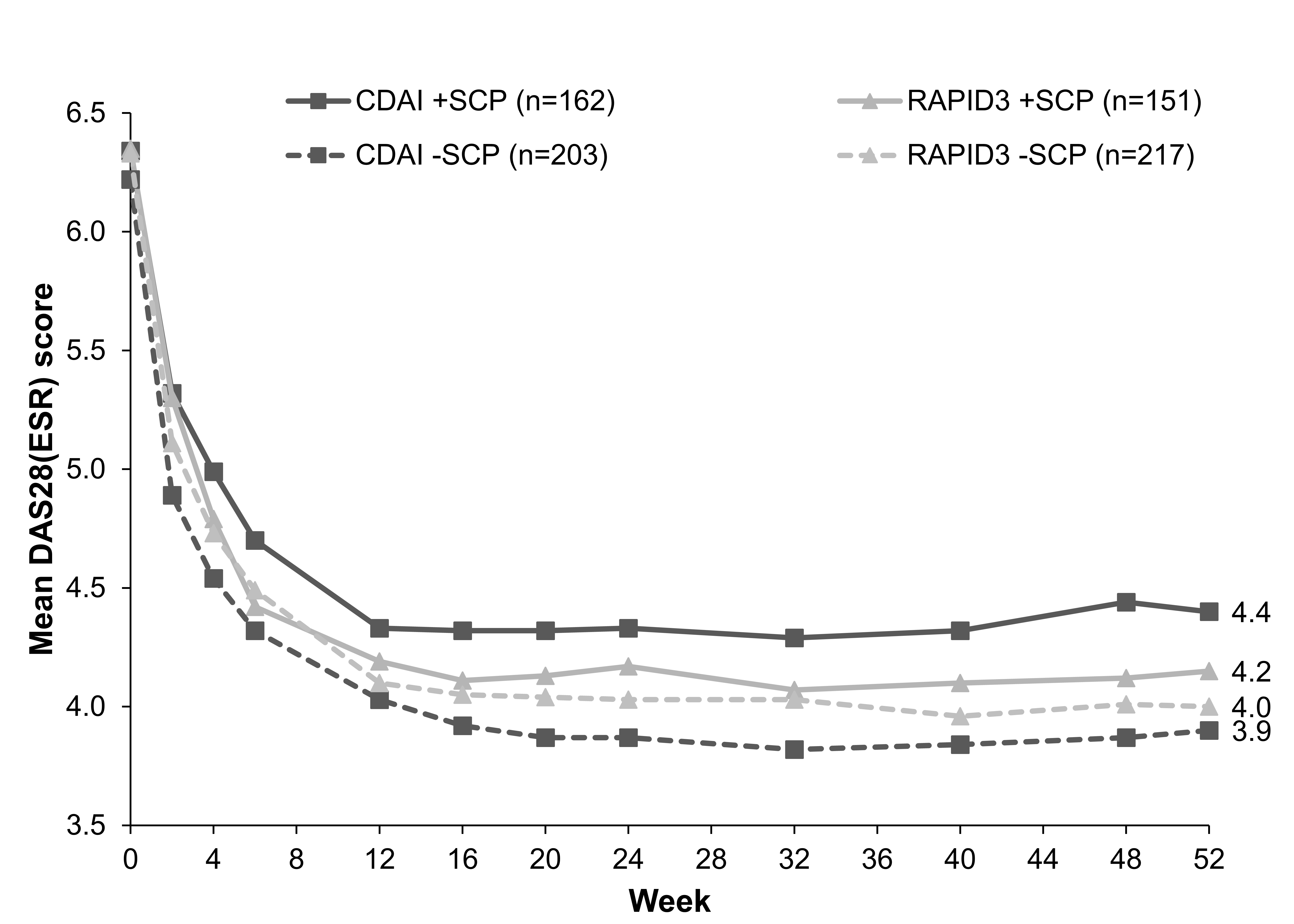
**

**Figure S1. Mean DAS28(ESR) score by SCP status (LOCF).** Full analysis set.
